# Explainable DCNN based chest X-ray image analysis and classification for COVID-19 pneumonia detection

**DOI:** 10.1038/s41598-021-95680-6

**Published:** 2021-08-09

**Authors:** Jie Hou, Terry Gao

**Affiliations:** 1 School of Biomedical Engineering, Guangdong Medical University, Dongguan, Guangdong China; 2 Counties Manukau District Health Board, Auckland, 1640 New Zealand

**Keywords:** Biological techniques, Computational biology and bioinformatics, Health care

## Abstract

To speed up the discovery of COVID-19 disease mechanisms by X-ray images, this research developed a new diagnosis platform using a deep convolutional neural network (DCNN) that is able to assist radiologists with diagnosis by distinguishing COVID-19 pneumonia from non-COVID-19 pneumonia in patients based on chest X-ray classification and analysis. Such a tool can save time in interpreting chest X-rays and increase the accuracy and thereby enhance our medical capacity for the detection and diagnosis of COVID-19. The explainable method is also used in the DCNN to select instances of the X-ray dataset images to explain the behavior of training-learning models to achieve higher prediction accuracy. The average accuracy of our method is above 96%, which can replace manual reading and has the potential to be applied to large-scale rapid screening of COVID-9 for widely use cases.

## Introduction

Coronaviruses are a large family of viruses that cause illness ranging from the common cold to more severe diseases, such as Middle East Respiratory Syndrome (MERS-CoV) and Severe Acute Respiratory Syndrome (SARS-CoV)^1^. This novel coronavirus (COVID-2019)^2–4^ is a new strain not previously identified in humans. A common clinical feature of severe COVID-19 infection is pneumonia^5–8^. Chest X-rays are a useful diagnostic tool for assessing various lung diseases, such as pneumonia, but the interpretation of the images can be challenging and time consuming by artificial^9,10^.

Part of the challenge is distinguishing between normal tissue and disease processes, a skill that must be learned through experience, particularly for some illnesses such as pneumonia where the difference is less obvious. With a great number of patients having chest X-rays taken as part of the diagnostic examination of suspected pneumonia each year at hospital alone, the evaluation of X-rays consumes a considerable number of resources.

Machine learning technology is currently being implemented in various sub-fields of medicine, including diagnostics, bioinformatics and educations. A convolutional neural network (CNN) is a deep machine learning algorithm^11^ that can be implemented in medical image classification and analysis to support speedy and correct decision making^12–15^. Also, computational models in IT and Intelligent Analytics have been significantly used in solving problems related to medical imaging^16,17^ and general COVID-19 healthcare monitoring systems^18,19^. Since the onset of the pandemic, many researchers have shown the effectiveness of using radiology images in identifying COVID-19 infection with various deep learning techniques. The general idea is that a set of medical images with category tags is used to train a deep learning CNN that is able to distinguish between noise and useful diagnostic information^20–23^. Recently, there has been a growing interest in developing COVID-19 diagnosis system by using deep learning techniques on well-known data sets used to train these networks^24–26^, also fusion LSTM network models and explicitly recurrent neural networks (RNN) are combined with CNN together to make superior predictions and to achieve promising performance. Islam MZ et al.^27^ introduced a combined deep CNN-LSTM network to estimate the uncertainty and interpretability in the detection of coronavirus; in their system, CNN was used for deep feature extraction and LSTM was used for detection using the extracted feature. The collection of 4575 X-ray images in the research, including 1525 images of COVID-19, were used as a dataset in this system, and the system achieved an accuracy of 99.4%. Prottoy Saha et al.^28^ developed an automated COVID-19 diagnosis from X-ray Images using CNN and Ensemble of Machine Learning Classifiers; in their research, a convolutional neural network was developed focusing on the simplicity of the model to extract high-level features from X-ray images of patients and binary machine learning classifiers (random forest, support vector machine, decision tree, and AdaBoost) were developed for the detection of COVID-19, and last these outputs were combined to develop an ensemble of classifiers, the method can achieve 98.91% accuracy. Islam et al.^29^ use a combined architecture of convolutional neural network (CNN) and recurrent neural network (RNN) to diagnose COVID-19 from chest X-rays, and gradient-weighted class activation mapping (Grad-CAM) was also used to visualize class specific regions of images that are responsible to make decision. All these are good methods for COVD-19 infection detection, but as combined different complex neural networks, it needs much more hardware resources and real-time processing is also a challenge issue.

The trained CNN is capable of interpreting new images by recognizing patterns that indicate certain diseases in the individual images. In this way, it imitates the training of a doctor, but the theory is that since it is capable of learning from a far larger set of images than any human, the CNN approach has more accurate results. Ghoshal et al.^3^ introduced a deep learning-based technique to estimate the uncertainty and interpretability in the detection of coronavirus. The authors have used a Bayesian Convolutional Neural Network (BCNN) and publicly available COVID-19 CXR images and found that the prediction uncertainty is extremely correlated with prediction accuracy. The performance results demonstrate an improvement in detection accuracy from 85.2% to 92.9% using pretrained VGG-16 model. They have also illustrated model interpretability by generating saliency maps to facilitate a better understanding of the results obtained by the proposed model. Narin et al.^5^ presented a transfer learning-based approach to the classification of CXR images into COVID-19 and normal categories. They have used three pretrained models such as InceptionV3, ResNet50, and InceptionResNetV2 in their system and achieved the highest 98% accuracy with ResNet50 for binary classification. However, the number of COVID-19 images in the curated dataset is only 50.

Singh et al. proposed an automated COVID-19 screening model^30^ is implemented by ensembling the deep transfer learning models such as Densely connected convolutional networks (DCCNs), ResNet152V2, and VGG16, and it has good performance in terms of accuracy, f-measure, area under curve, sensitivity, and specificity, but the sample is mainly for CT images. Singh et al. also developed an automated analysis method^31^ which can save the medical professionals’ valuable time, and the method outperforms the competitive machine learning models in terms of various performance metrics without needing a lot of training sets. Gianchandani et al. proposed two different ensemble deep transfer learning models^32^ which have been designed for COVID-19 diagnosis utilizing the chest X-rays. Both models have utilized pre-trained models for better performance, and can differentiate COVID-19, viral pneumonia, and bacterial pneumonia.

A pilot study using publicly available chest X-rays of no pneumonia patients and patients with coronavirus should promise in that it is possible to train a CNN to distinguish between these two groups with approximately 90% high accuracy^33^. In addition, there is the potential to distinguish viral from bacterial pneumonia, which is particularly relevant to COVID-19 infection because pneumonia is directly associated with the virus rather than a bacterial complication. A Bayesian Convolutional Neural Network (BCNN) technique for estimating infection of coronavirus was introduced^34^. The accuracy of the method is around 90%. A transfer learning approach was used to classify the CXR images for COVID-19 detection, and the CNN models including InceptionV3, ResNet50, and InceptionResNetV2, however, the samples of COVID-19 images were very limited, and the performance was influenced^35^.

The research content of this paper is that a set of X-ray medical lung images with category tags used to train a two tiers CNN called DCNN, which can distinguish normal, coronavirus infection, COVID-19 and other virus infection. We have proposed a system which combines two deep learning based CNN model that exploits the benefit of a weighted average of the model weights. The first CNN is used for (Normal, infected by bacteria, infected by Virus) detection, and the outputs (infected by Virus) of the first Convolutional Neural Network are used as input of the second Convolutional Neural Network to obtain a robust classification of these images into COVID-19, normal, and other virus pneumonia categories. Such a tool could increase the speed and accuracy of interpreting and thereby improve the overall treatment of patients, also reduce the resource demanding which is useful for COVID-19 disease detection.

## Design and methods

### Study design and type

This research built a diagnostic system that uses open-to-public coronavirus infector chest X-ray images from^36^ for training. The historical data are split into a training and a validation set, which are composed of three samples of normal, coronavirus infection and virus infection. The CNN is then trained on the training set and the predictive value of the tool, once trained, and determined by using the validation set. Tests of what parts of the images by which the CNN uses to determine the output are explored to ensure that the output is clinically relevant. After this initial analysis, an extraction of massive texture features was applied and can serve to provide additional information for the diagnosis of COVID-19.

### Participants

The training of the CNN needs to have non pneumonia and pneumonia representing X-rays that are alike in all other aspects that may influence how an X-ray looks, so the deep CNN is trained to look at the actual difference based on the presence of pneumonia and no other factors associated with pneumonia. Since a patient who is diagnosed and treated in-house has at least an X-ray to diagnose the condition and an X-ray to confirm that the pneumonia is gone, we have X-rays from the same patient with and without pneumonia. For this reason, the training set is a random selection of patients. The chest X-rays used are the first X-ray taken of the patient during the admission from the moment a pneumonia was suspected, and the last X-ray taken before discharge. Both X-rays taken used the same position of the patient (standing/lying in bed).

### Platform design and the outcomes

For each record in the validation set, the following outcomes are collected:

- Diagnosis as determined by the trained CNN
- Gold standard diagnosis, as determined by radiologist and confirmed by discharge department.
- If an X-ray as determined not to show pneumonia but clinical diagnosis shows pneumonia, then at least one other X-ray of that admission episode should have shown pneumonia. If either of these two criteria is not met, then the X-ray is determined by showing pneumonia.
- If an X-ray was determined to show pneumonia, then the clinical diagnosis result had to show pneumonia; otherwise, it was determined not to show pneumonia.

### Sample size calculations

The training data size depends on the complexity of the CNN model, such as the number of inputs/outputs, the relationships between parameters, the noise in the data, and the variance and standard deviation of every parameter, so the best approach is to ensure that our data cover all the ranges we want for all parameters. Normally, the number of samples is at least 10 times more than the number of CNN training parameters, so we initially set the training samples to approximately 1400 chest X-ray images, which include 400 normal images, 400 pneumonia infected by bacteria images, 400 pneumonia infected by other virus images, and 200 pneumonia infected by COVID-19 images. Testing samples are 400 chest X-ray images (100 images for each class). 100 chest X-ray images are used for validation, which also include 50 COVID-19 infection images.

### Ethics approval and consent to participate

The research is approved by Guangdong Medical University and CMDHB. We only use the historical on-line data and X-ray images. There will be no interaction with or impact on patients.

## Implementation procedure

### The theoretical basis of the algorithm

**Figure 1:**
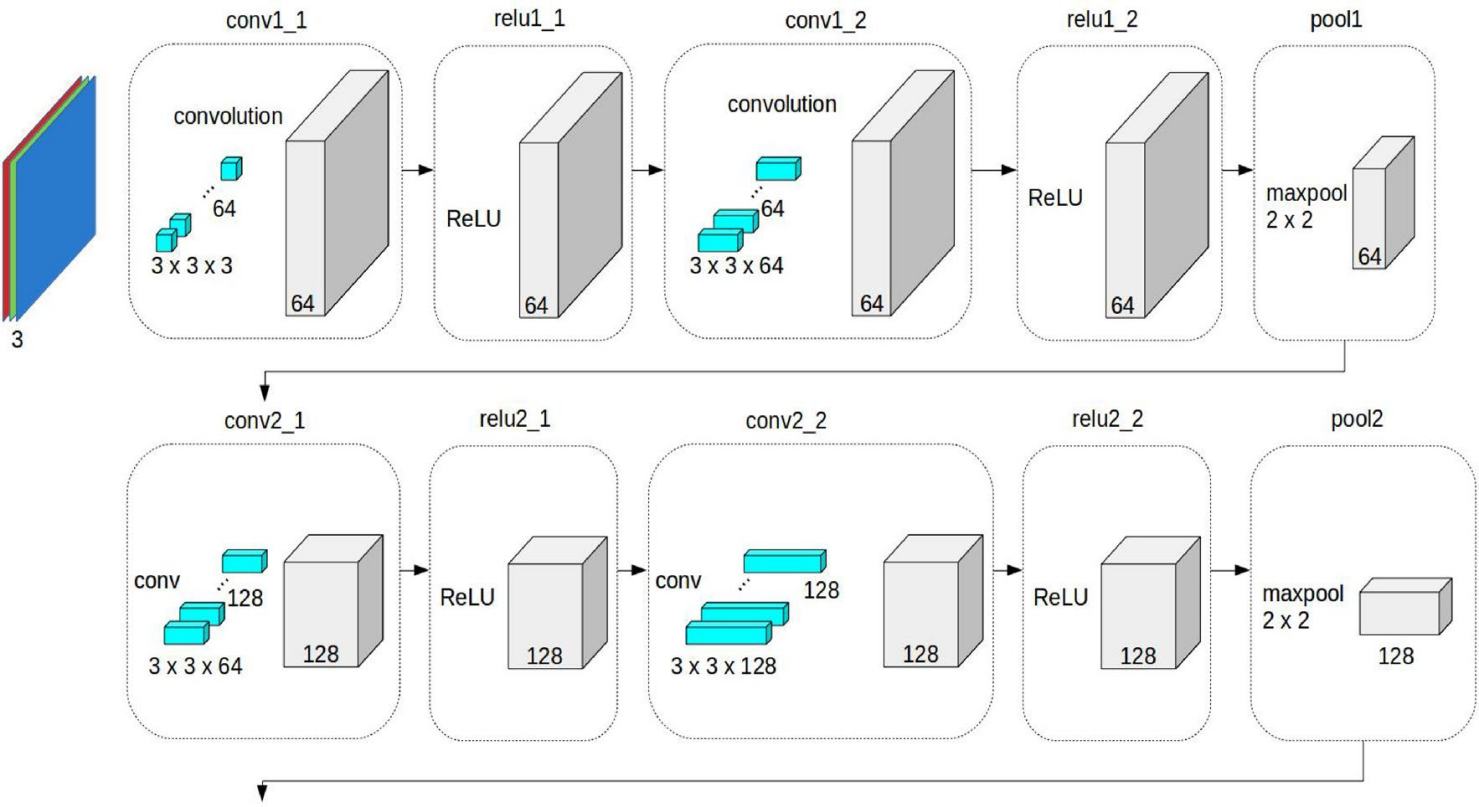
VGG-19 structure.

The primary step of this research is a deep CNN designed and trained to assist radiologists with diagnosis by distinguishing COVID-19 pneumonia from non-COVID-19 pneumonia in patients at hospital with high predictive values using clinically relevant parts of the images. Then, this deep CNN is used to distinguish bacterial from viral pneumonia amongst those patients with pneumonia at hospital with high predictive values using clinically relevant parts of the images. The CNN is designed based on VGG-19^37^ which is a variant of VGG model which in short consists of 19 layers (16 convolution layers, 3 Fully connected layer, 5 MaxPool layers and 1 SoftMax layer as in Fig. 1 and reduced the levels, changing the convolutional kernels to make it more feasible.

The new structure of DCNN is designed as: let *x* be set as the input vector, ${\varphi }_{i}$ is the radial basis function, $N$ is the number of input training samples, and $y$ is the output of the neural network, a new method which measures the similarity between the test images and the ground truth images to improve the detection robust of the system.

1 $$y=W(N)\phi (x(N))$$

Then, $d(n)$ is the output response of the $n$ iterations of the neural network, and the error is defined as:

2 $$e\left(n\right)=d\left(n\right)-y\left(n\right)=d\left(n\right)-W\left(n\right)\phi (x(n))$$

and the objective function as:

3 $$J\left(n\right)=\frac{1}{2}{e}^{2}\left(n\right)=\frac{1}{2}{[d\left(n\right)-W\left(n\right)\phi \left(x(n\right))]}^{2}$$

and to the weight is updated according to:

4 $$\Delta w\left(n+1\right)=\left(n+1\right)-\Delta w\left(n\right)$$

According to the gradient descent with the momentum algorithm, I can obtain:

5 $$\Delta w\left(n+1\right)={\gamma }_{n}\Delta w\left(n\right)-\eta {}_{c}{D}_{w(n)}^{\alpha }J(n)$$

where $\eta$ is the learning rate, $0<\alpha <\mathrm{1,0}<\gamma <\eta$, $\gamma$ is the momentum factor, and ${\gamma }_{n}$ is the momentum coefficient designed as follows:

6 $${\gamma }_{n}=\left\{\begin{array}{cc}\frac{\gamma \Vert {}_{c}{D}_{w(n)}^{\alpha }J(n)\Vert }{\Vert \Delta w\left(n\right)\Vert }& \Vert \Delta w\left(n\right)\Vert \ne 0\\ 0& \mathrm{else}\end{array}\right.$$

$||\cdot ||$ is the Euclidean normalization. According to the definition of the fractional derivative, the output result as:

7 $$y={\sum }_{j=1}^{N}{J}_{j}^{^{\prime}}(w\phi \left({x}_{j}\left(n\right)\right))\phi ({x}_{j}\left(n\right))\frac{{{(w}_{i}\left(n\right)-c)}^{1-\alpha }}{\left(1-\alpha \right)\Gamma (1-\alpha )}$$

For given parameters *a*, *b*, and *c* in CNN model, the neural network is used to obtain the enhanced image by searching the optimal objective function *E*:

8 $$E={\Vert IR-S\Vert }_{2}^{2}+a\left\| \frac{{\nabla }_{x}I}{\frac{\sum_{\Omega }{\nabla }_{x}I}{\left|\Omega \right|}+\varepsilon }\right\|+b\left\| \frac{{\nabla }_{y}I}{\frac{\sum_{\Omega }{\nabla }_{y}I}{\left|\Omega \right|}+\varepsilon }\right\| +c\left({\Vert {\nabla }_{x}R\Vert }_{1}+{\Vert {\nabla }_{y}R\Vert }_{1}\right)+\lambda {\Vert I-\mathrm{max}\left(\mathrm{max}{S}^{c}\right)\Vert }_{2}^{2}$$

Then the class similarity is computed as an image quality evaluation index, which measures the similarity between the test images and the ground truth image:

9 $$SSIM(x,y)=l(x,y{)}^{\lambda }u(x,y{)}^{\beta }s(x,y{)}^{\mu }$$

### The proposed algorithm: DCNN

**Figure 2:**
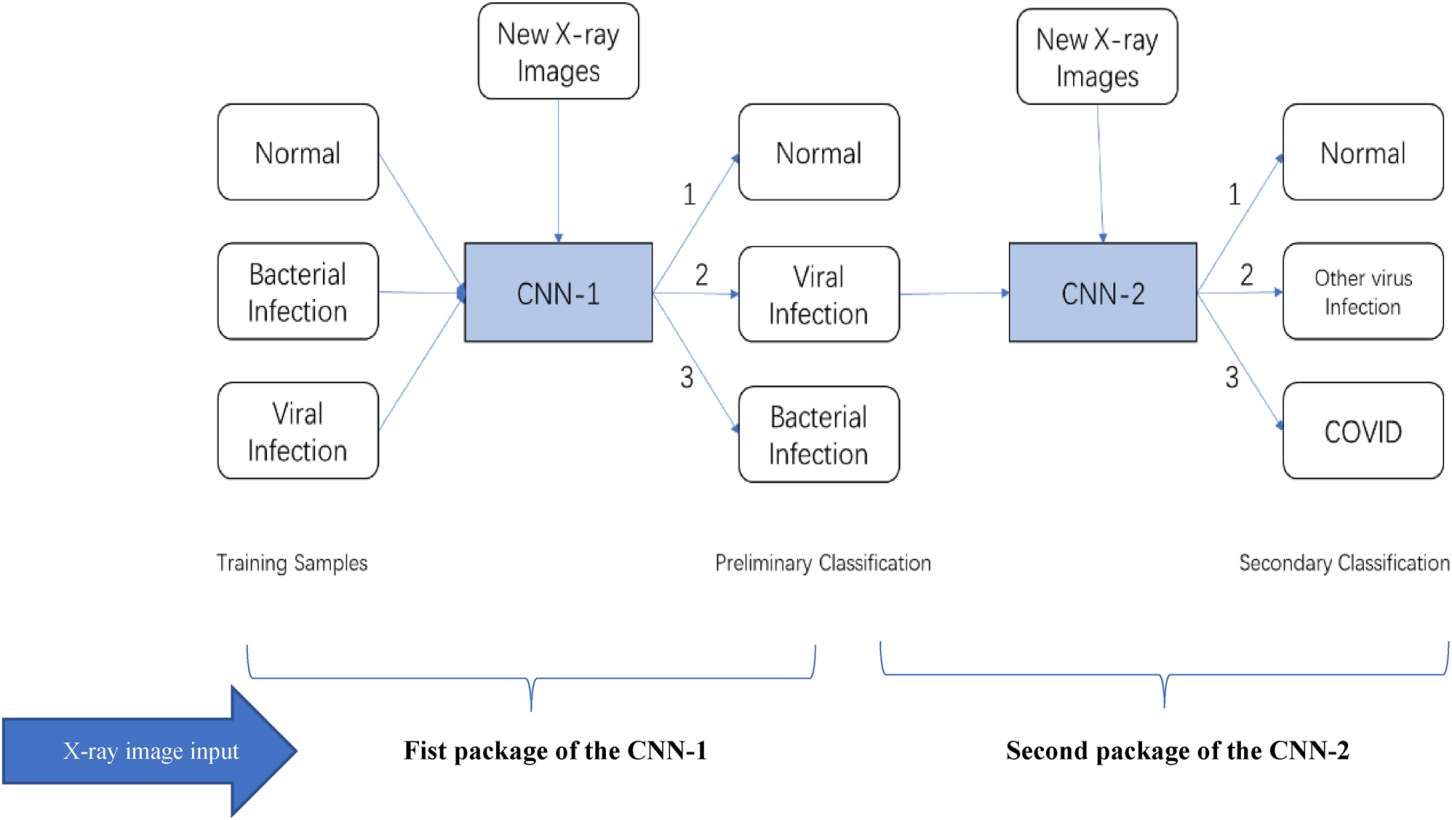
The execution framework of the proposed DCNN.

Based on the above application background and theoretical basis, this section introduces the proposed DNN framework, which is shown in Fig. 2. The DCNN is divided into two training levels. One innovation of our system is that two separate CNN are used for different categories detection, and the input of the second CNN is form one stream output of the first CNN.

- First, the first CNN-1is trained by inputting the training samples with category labels, all the unknown parameters of CNN-1 are obtained, and the CNN-1 is valid by the validation data set.
- Then, CNN-1 is used to determine the test samples to separate out the standard set, the virus infection set and the bacterial infection set.
- Third, the virus infection output set is labelled for the CNN-2, which has three categories of normal, COVID-19 and other virus infection. So, the second CNN-2 is trained to obtain all the unknown parameters and the CNN-2 is valid by the validation set; Finally, CNN-2 is used to determine the final test samples to separate out the standard set, other virus infection set, and COVID-19 set.

### Training the DCNN

The convolution layers have a hierarchical structure and are core building blocks of a CNN^38^. The DCNN applying individual network levels and rapid combinations of features take place before the forecasting stage. The input of the first convolution layer is the input space, and the output is the feature map. The input and output of the next convolutional layers are feature maps of the input space. The number of convolutional layers is set by the programmer. The set of feature maps is obtained as the output of convolutional layers. The complex features of the input space are represented by using the stacked hierarchical structure of convolutional layers. The obtained features from the convolutional layers are fed to the pooling layer. An activation function such as ReLU is applied to the obtained feature map. In this layer, the relevant features are retained, and the rest are discarded. A dropout layer with a dropout factor of 0.5 has also been used for the regularization of the model. Then, the feature maps of the corresponding depths of the contraction path are fed as input. The obtained features are transformed into a one-dimensional array called the feature vector. The feature vector is a one-dimensional array and is the input for the fully connected layer. The fully connected layer calculates the output of the CNN.

The regression branch predicts the distances from the center of each grid to the four sides of the bounding box. Centeredness is a coefficient in the range of [0,1] for each grid. The farther the grid center is from the object center, the smaller the coefficient is. The centeredness and class are multiplied and then serve as the input of nonmaximal suppression (NMS). The sclera block is similar to a fully convolutional network (FCN), where the input feature map is unsampled 4 times to obtain a score map. After these operations using the ReLU activation function, the nonlinear transformation of signals is performed for each matrix. The obtained results are sent to the pooling layer. In this layer for each cell, the max (or average) pooling operations are performed. In the pooling layer, down sampling operations have been performed to reduce the size of the feature matrix derived from the convolution layer. After training, the class index is used to measure the class activation map, and the layers can be used when visualizing the class activation map. The CNN gradient model is constructed by supplying the inputs of the pretrained model and the output of the final layer in the network. The average of the gradient values is computed by using connection Weights, and the ponderation of the filters is computed with respect to the Weights, so the connection heatmap can be formed and normalized such that all values are set in the range [0, 1], and the resulting values can be scaled to the range [0, 255] to finally show the regions of interest with bright color that can be used for medical purpose analysis. Details are as in Table 1. Also, the results from the output categories can then be averaged (or otherwise combined) to produce a single estimation, and the advantage of this method is that all observations are used for both training and validation, and each observation is used for validation exactly once.

**Table 1:** The parameters of each level for CNN.

	Layer Type	Output Shape	Param
Input Layer	Input	64 × 64 × 3	0
Hidden Layer 1	Conv1	64 × 64 × 32	896
	ReLU	64 × 64 × 32	0
	Pool1	32 × 32 × 32	0
Hidden Layer 2	Conv2	32 × 32 × 64	18,496
	ReLU	32 × 32 × 64	0
	Pool2	16 × 16 × 64	0
Hidden Layer 3	Conv3	16 × 16 × 128	73,856
	ReLU	16 × 16 × 128	0
	Pool3	8 × 8 × 128	0
Classification layer	Flatten	8192	0
	Dense1	16	131,088
	ReLU	16	0
	Dense2	64	1088
	ReLU	64	0
	Dense3	128	8320
	ReLU	128	0
	Dense4	2	258

The first convolutional layer learns 32 convolutional filters, each of which is $3\times 3$. Then, rectified linear units (ReLUs) are applied as an activation function that has output 0 if the input is less than 0 and output otherwise. The following layers use similar processing. The fully connected layer uses SoftMax for the activation function.

As tuning the hyperparameters of CNN is very critical in obtaining a high-accuracy model, “grid search”^39^ by sampling various hyperparameters from a distribution is used in the CNN. It starts by defining a set of all hyperparameters and the associated values which want to be explored, then examines all combinations of these hyperparameters. For each possible combination of hyperparameters, a model is trained on them and the hyperparameters associated with the highest accuracy are then returned. The learning rate grid is [0.1, 0.01, 0.001], Dropout rate grid is [0.2, 0.3, 0.5], Batch size grid is [8, 32, 64], and Epoch’s grid to train is [60, 120, 180].

### Testing the DCNN

Once the CNN has been trained using the training set, it is used to diagnose all the X-rays in the test set. For each case, the proportion of each diagnosis can be obtained. The parameters used to indicate the performance of CNN are as accuracy, re-call, and F1 score.

**Accuracy**: The ratio of correct predictions (true positives + true negatives) to the total number of predictions.

**Recall**: The fraction of the cases classified as positive that are actually positive (the number of true positives divided by the number of true positives plus false negatives).

**F1 Score**: An overall metric that essentially combines precision and recall.

The testing rules used are as follows:

1. Positive and predictive values are determined with family-wide 95% confidence intervals (Bonferroni correction) for the output of the CNN in determining COVID-19 pneumonia.
2. Positive and predictive values are determined with family-wide 95% confidence intervals (Bonferroni correction) for the output of the CNN in determining bacterial against viral pneumonia.

Examples of the type of X-ray data that is analyzed using our DCNN approach is shown in Fig. 4. The test images are from Ref^36^. and “The New England Journal of Medicine, 2020: January 31. DOI: 10.1056/ NEJMoa 2001191”^40^. The data augmentation methods applied in the proposed DCNN are scale, shift, rotate, salt and pepper noise, and flip (Processes are as: To use a batch of images used for training, and take this batch and applies a series of random transformations to each image in the batch. Replaces the original batch with the new, randomly transformed batch. Trains the CNN on this randomly transformed batch.). By applying these small transformations to images during training, variety in the training dataset has been created and improves the robustness of the proposed model. Generators are implemented for dynamic augmentation of the input image and generation of the corresponding ground truth labels.

**Figure 3:**
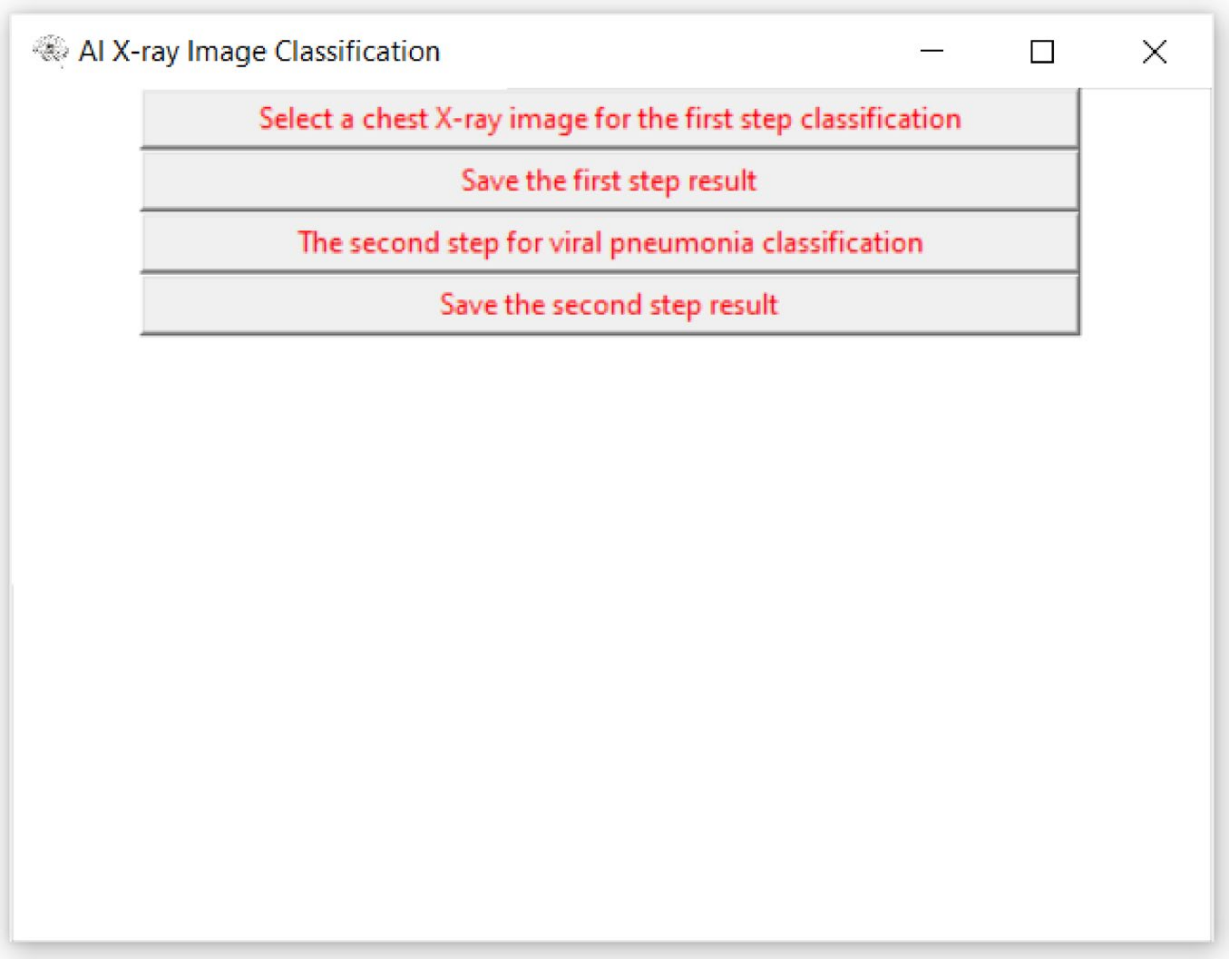
User interface (UI) of the system.

**Figure 4:**
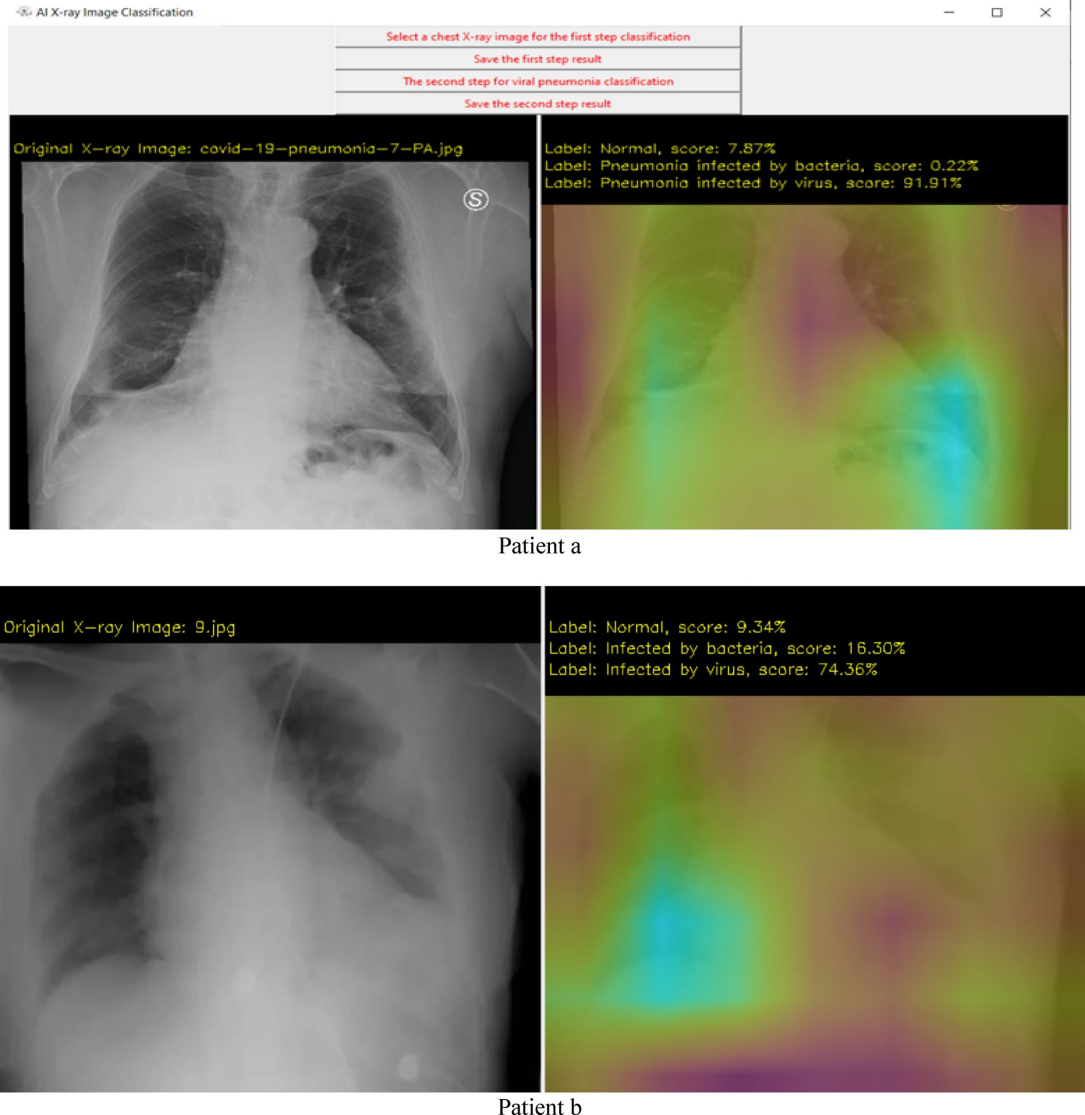

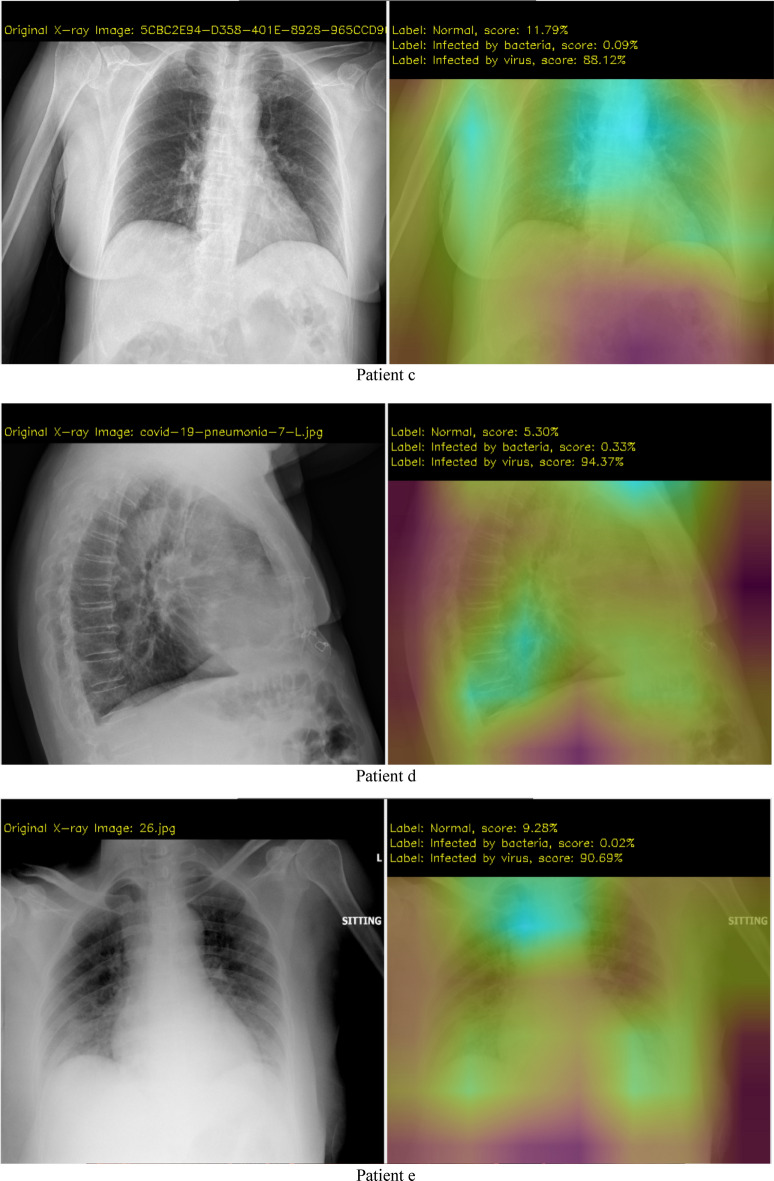
First step results.

**Figure 5:**
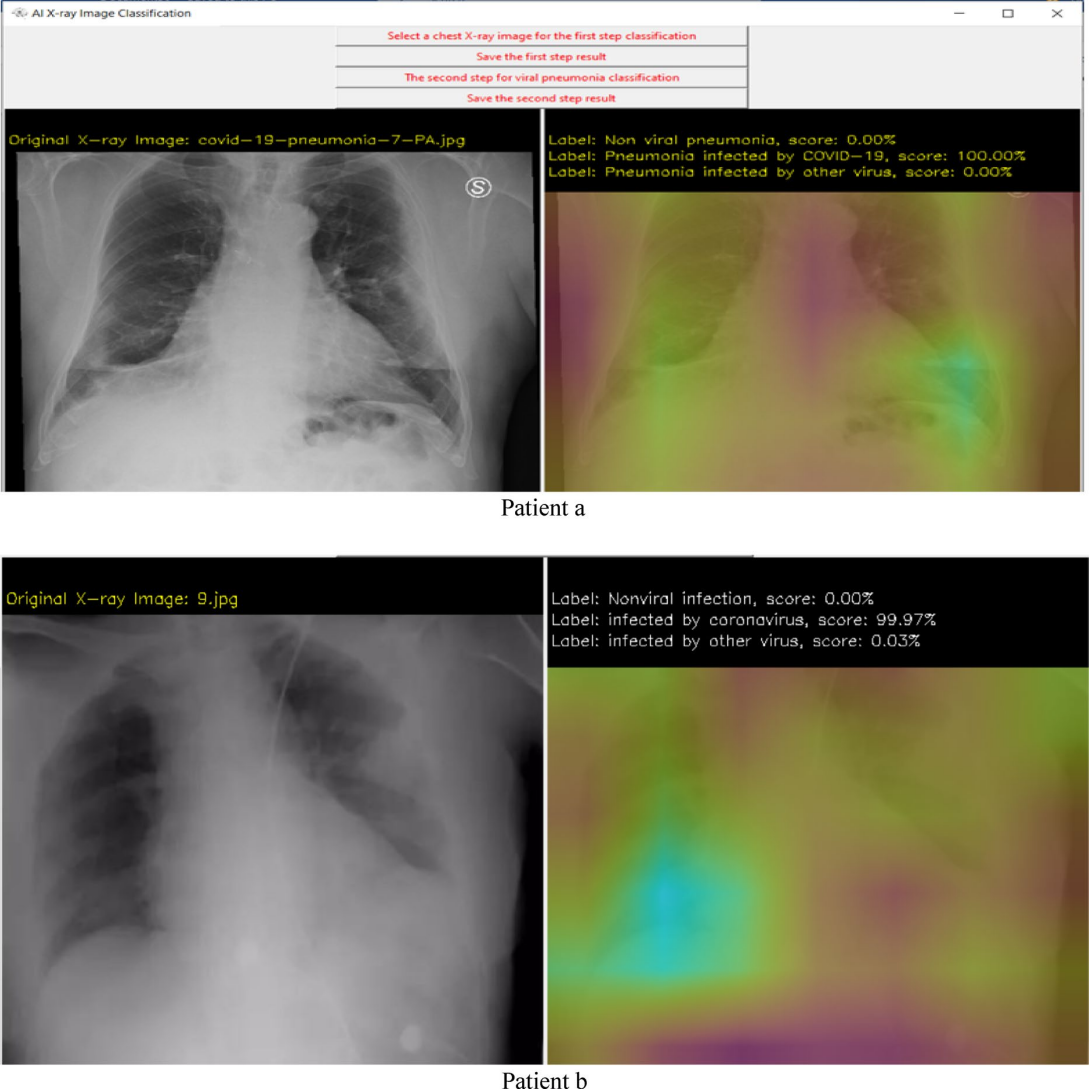

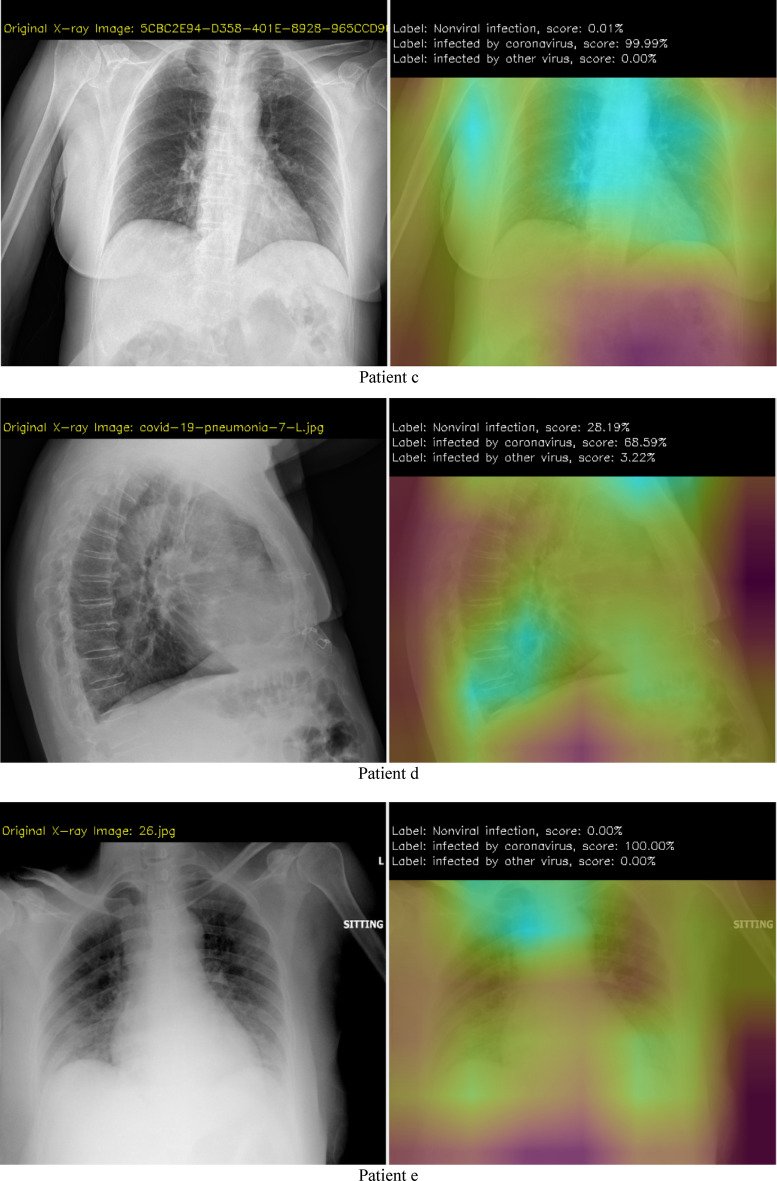
Analysis results demo.

The experiment and software are based on TensorFlow 2.1-GPU^41^, Python 3.7^42^ and CUDA 10.1^43^ for accelerated training. The hardware for this system includes two i7-CPUs, 16.0 GB memory, and a 500 GB SSD drive, NVIDIA GeForce GTX 1660 Ti GPU, and it takes approximately 4 h of training to converge. The user interface of the system developed in this research is shown in Fig. 3. Figures 4 and 5 show the detection results. From the results we can see that the system is very robust for different X-ray images of body position, angle, gender, and size.

Results from step 1 (the first CNN). The left image shows the original X-ray data, the right image shows the possibility of 3 different cases (normal, infected by bacteria, infected by virus), and the magenta color shows which part has the potential infection problem.

Results from step 2 (the second CNN). If step 1 shows viral pneumonia with the highest score, continuous processes are analyzed and checked if the patient has COVID-19 infection or not. The right image shows the possibility of 3 different cases (non-viral pneumonia, infected by COVID-19, infected by other viruses), and a brighter color shows which part has the potential infection problem.

**Figure 6:**
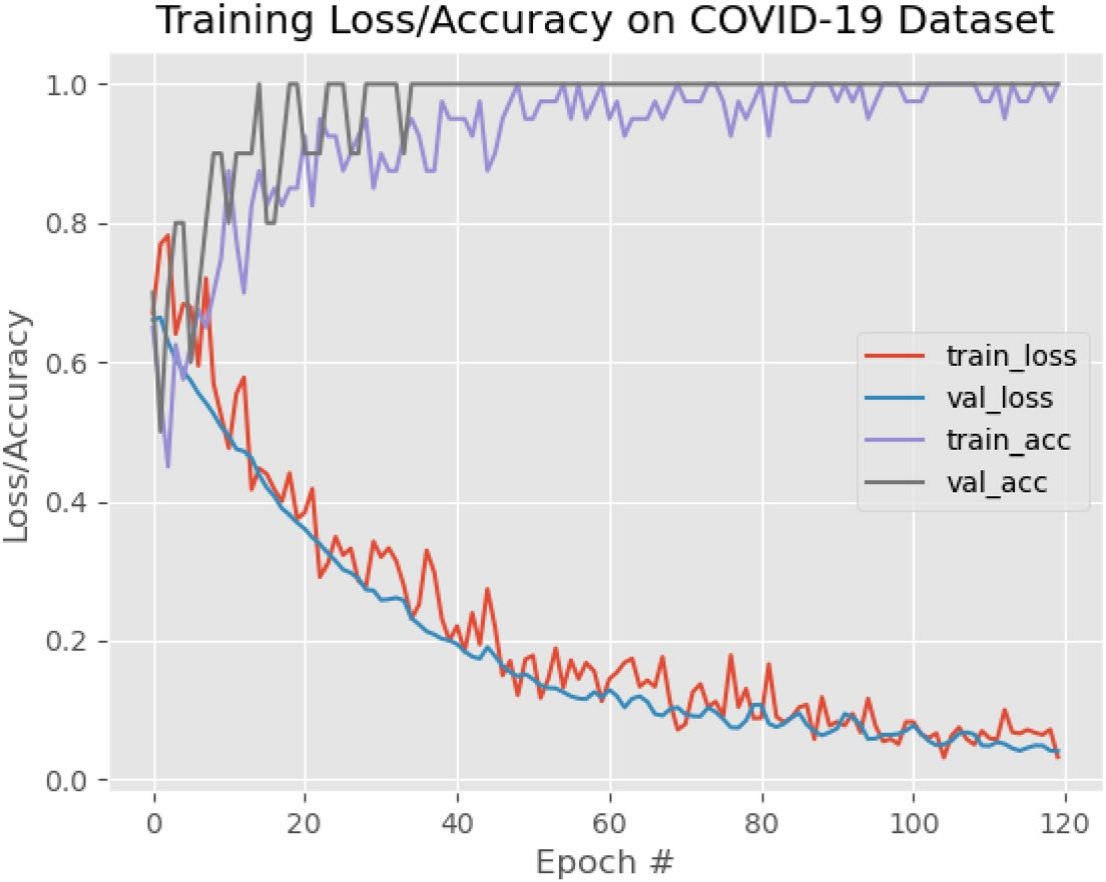
Loss/accuracy graph.

The architecture of DCNN categorizes benefits in X-ray medical imaging, such as the number of modules in interconnected operations and input modalities, dimension in input patch, quantity of time predictions and contextual information about implicit and explicit. The test results of the proposed DCNN are shown in Table 2 and the training loss and accuracy in Fig. 6 also indicate the robust learning of our model.

**Table 2:** Experimental results for test database.

X-ray Category	Parameters			
	Precision	Recall	F1-score	Support
Normal	0.97	0.94	0.95	572
Pneumonia infected by Bacteria	0.99	0.93	0.96	213
Pneumonia infected by COVID-19	0.96	0.98	0.97	117
Pneumonia infected by normal Virus	0.98	0.96	0.97	410
Average	0.97	0.95	0.96	1312

The performance results of different CNN models (ResNet50V2^44^, InceptionV3^45^, VGG-16^46^, VGG-19^37^, DenseNet^47^) are compared by using our COVID-19 dataset, as shown in Table 3. For comparison testing, 300 chest X-ray images (100 images for each class) are used, learning rate is initialized as 0.001, epoch is set 120, and batch size is used as 8.

**Table 3:** Performance results of different models for validation database.

Model	Class	Accuracy %	Precision %	F1-score %
ResNet50V2	Pneumonia by bacteria	90.0	93.0	92.0
	Pneumonia by normal virus	94.20	94.0	94.0
	COVID-19	91.25	91.0	91.0
InceptionV3	Pneumonia by Bacteria	86.50	90.0	88.0
	Pneumonia by Normal Virus	84.20	86.0	85.0
	COVID-19	80.30	80.60	80.0
VGG-16	Pneumonia by bacteria	87.0	91.0	89.0
	Pneumonia by normal virus	93.40	90.0	92.0
	COVID-19	81.38	81.75	81.0
VGG-19	Pneumonia by bacteria	84.0	89.0	86.0
	Pneumonia by normal virus	94.5	91.0	92.8
	COVID-19	86.0	87.0	86.5
DenseNet	Pneumonia by bacteria	86.0	91.0	89.0
	Pneumonia by normal virus	93.5	90.0	91.8
	COVID-19	88.0	87.0	87.5
Our Model	Pneumonia by bacteria	99.0	99.0	99.0
	Pneumonia by normal virus	98.15	96.20	97.0
	COVID-19	96.03	96.15	96.0

**Figure 7:**
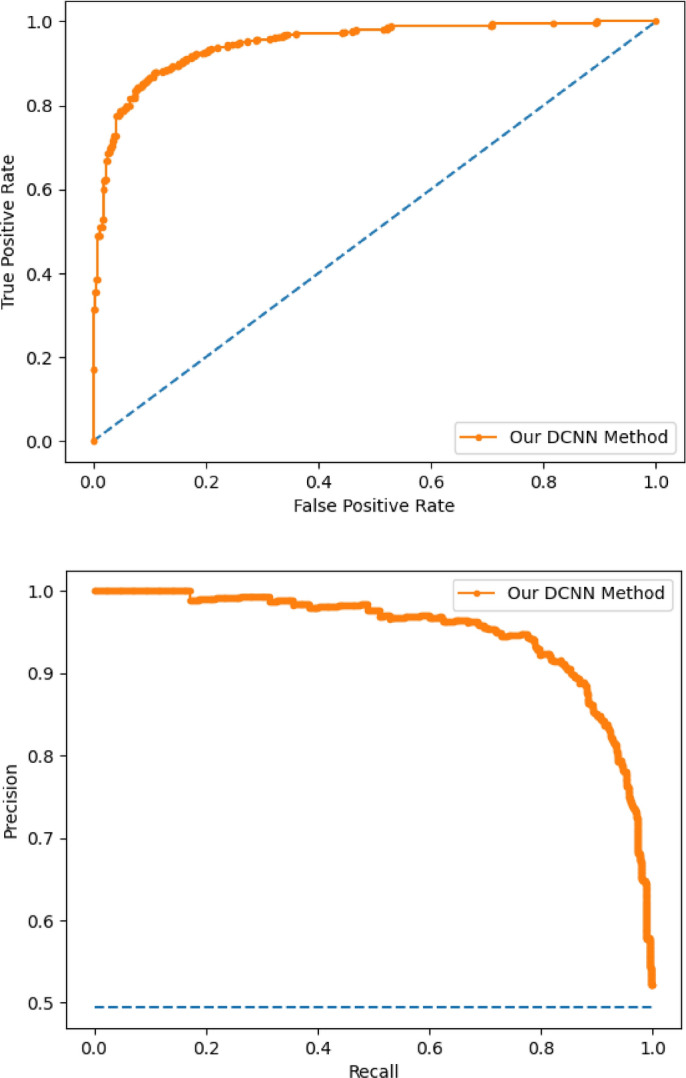
ROC and precision-recall curve.

Figure 7 shows the performance of the evaluated model which includes the receiver operating characteristic (ROC) and precision-recall curve. A ROC curve plots the true positive rate (TPR) against false positive rate (FPR), and the model shows superior discrimination abilities for COVID-19 classes. The precision-recall plot shows the tradeoff between recall and precision for various threshold levels.

## Conclusions

To speed up the discovery of disease mechanisms, this research developed a two-level deep CNN-based chest X-ray classifier system to detect abnormalities and extract textural features of the altered lung parenchyma that can be related to specific signatures of the COVID-19 virus. Explainable AI explanations method especially the proposed DCNN can help to construct more robust model and it especially helps doctors to understand complex data distributions such as X-ray images which play an important role in the diagnosis of COVID-19 infection from other pneumonia as advanced imaging evidence. As example-based explainable methods work well if the feature values of an instance carry more context, meaning the data has a structure, like images, so artificial intelligence (AI) algorithms and radionic features derived from chest X-rays can be of great help to undertake massive screening programs that could take place in any hospital with access to X-ray equipment and aid in the diagnosis of COVID-19. The designed system has 96.03% accuracy, 96.15% precision, and 96.0% recall for COVID-19 X-ray images (including different body position, angle, size, and gender). Although the results were promising, further investigation is needed on a larger dataset of COVID-19 images, to have a more comprehensive evaluation of accuracy rates. The performance of this system can also be improved by using more robust optimization techniques such as: Elephant herding optimization (EHO), Bull optimization algorithm (BOA), Parliamentary optimization algorithm (POA), and Bumble bees mating optimization (BBMO), which are also included in our future works. What is more, the similar system can be used for other kind of medical image data, such as CT, MRI, MEG, et al., helping for different fields of early diagnosis.

## Abbreviations

AI: Artificial intelligence
BCNN: Bayesian convolutional neural network
CNN: Convolutional neural network
COVID: Coronavirus disease
CXR: Chest x-ray
DCNN: Deep convolutional neural network
FCN: Fully convolutional network
GPU: Graphics processing unit
NMS: Nonmaximal suppression
ReLUs: Rectified linear units
