# Author Correction: Explainable DCNN based chest X-ray image analysis and classification for COVID-19 pneumonia detection

**DOI:** 10.1038/s41598-021-98624-2

**Published:** 2021-09-20

**Authors:** Jie Hou, Terry Gao

**Affiliations:** 1 School of Biomedical Engineering, Guangdong Medical University, Dongguan, Guangdong China; 2 Counties Manukau District Health Board, Auckland, 1640 New Zealand

Correction to: *Scientific Reports* https://doi.org/10.1038/s41598-021-95680-6, published online 09 August 2021

The original version of this article contained errors.

In the Design and methods section, under the subheading 'Ethics approval and consent to participate',

"The research is approved by Guangdong Medical University and Middlemore hospital super clinic office. We only use the historical on-line data and X-ray images. There will be no interaction with or impact on patients."

now reads:

"The research is approved by Guangdong Medical University and CMDHB. We only use the historical on-line data and X-ray images. There will be no interaction with or impact on patients."

The original article has been corrected.

